# TaCML49-B, a Calmodulin-like Protein, Interacts with TaIQD23 to Positively Regulate Salt Tolerance in Wheat

**DOI:** 10.3390/plants14203163

**Published:** 2025-10-15

**Authors:** Jingna Ru, Jiamin Hao, Bingqing Hao, Xiaoqian Ji, Jiale Yang, Hongtao Wang, Baoquan Quan, Pengyan Guo, Jiping Zhao, Huawei Shi, Zhaoshi Xu

**Affiliations:** 1 The Industrial Crop Institute, Key Laboratory of Sustainable Dryland Agriculture of Shanxi Province, Shanxi Agricultural University, Taiyuan 030031, China; 2 Agricultural College, Shanxi Agricultural University, Jinzhong 030810, China15076316832@163.com (X.J.);; 3 State Key Laboratory of Crop Gene Resources and Breeding, Institute of Crop Sciences, Chinese Academy of Agricultural Sciences (CAAS), Beijing 100081, China; xuzhaoshi@caas.cn

**Keywords:** wheat, calcium signaling, calmodulin-like, salt stress

## Abstract

Calcium signaling is essential for coordinating plant responses to diverse stimuli and regulating growth and development. Among calcium sensors, calmodulin (CaM) and CaM-like proteins (CMLs) represent a class that, despite increasing research, remains incompletely characterized in wheat, with many interacting partners and biological functions remaining largely elusive. This study conducted bioinformatics analyses of subgroup II CaM/CMLs, characterizing their phylogenetic relationships, conserved motifs, sequence features, and *cis*-elements. Expression analysis revealed that *TaCML49-B* was significantly upregulated in roots under salt stress. Moreover, TaCML49-B was localized to nucleus, cytoplasm, and membrane. Function characterization demonstrated that overexpression of *TaCML49-B* in *Arabidopsis* enhanced salt tolerance, whereas the BSMV-VIGS silencing of *TaCML49-B* reduced salt resistance in wheat. Furthermore, STRING database prediction analysis and bimolecular fluorescence complementation (BiFC) assay confirmed that TaCML49-B can physically interact with TaIQD23, which encodes an IQ67 domain protein, suggesting its potential involvement in the salt stress signaling pathway. Collectively, our findings indicate that *TaCML49-B* functions as a positive role in wheat salt stress response, thereby providing novel insights into the functions of TaCML genes and calcium signaling in wheat.

## 1. Introduction

As a universal second messenger, calcium (Ca^2+^) plays pivotal roles in plant growth, development, and stress signal transduction [1]. Calmodulin (CaM) and CaM-like proteins (CMLs) are the primary calcium sensors in eukaryotes [2]. By binding Ca^2+^ through characteristic EF-hand motifs, these proteins undergo conformational changes and regulate the activity of multiple downstream effectors in response to Ca^2+^ signaling [3]. Most CMLs contain two, four, or six EF-hand domains but lack other recognizable functional motifs, and they typically share at least 16% amino acid sequence identity with canonical CaMs [4].

CaM/CMLs interpret intracellular Ca^2+^ signatures and modulate diverse physiological and stress-related processes through interactions with specific target proteins [5]. Functional studies across different plant species have highlighted the critical roles of CaM/CMLs in development and stress adaptation [6]. For example, in *Arabidopsis*, *CML16* and *CML15* display distinct promoter activities during development [7], while *CML41* contributes to bacterial defense by mediating Ca^2+^-dependent signaling specificity [8]. In tobacco, the CaM isoform *rgsCAM* (regulator of gene silencing, CaM) enhances viral resistance by inhibiting the activity of the helper component protease HC-Pro [9]. Overexpression of *ShCML44* promotes seed germination and seedling vigor [10], and *CML10* regulates ascorbic acid synthesis to enhance tolerance to drought, ozone, and UV stress [11]. Similarly, *CML14* undergoes structural changes upon Ca^2+^ binding, which stabilizes the protein and modulates its functional specificity [12]. In rice, *OsCML16* is transcriptionally regulated by *OsERF48*, thereby promoting root growth and drought tolerance [13]. In contrast, heterologous expression of *GsCML27* reduces salt tolerance by disrupting ion homeostasis and osmotic regulation [14]. In wheat, several *TaCML* genes, including *TaCML17*, *TaCML21*, *TaCML30*, *TaCML50*, *TaCML59*, and *TaCML75*, are implicated in responses to abiotic stresses [15]. Moreover, overexpression of *TaCML20* enhances the accumulation of water-soluble carbohydrates and improve yield-related traits under stress conditions [16]. *TaCML36* has been reported to positively contribute to the wheat immune response against *Rhizoctonia cerealis* by modulating the expression of defense-associated genes [17]. In addition, *Arabidopsis* overexpressing *TaCAM2-D* exhibited enhanced tolerance to both drought and salt stress. TaCAM2-D was further verified to interact with TaMPK8, supporting the functional involvement of *TaCAMs* in wheat stress responses [18]. These findings collectively highlight the functional diversity of CaM/CMLs and their importance in mediating plant adaptation to environmental stress.

IQ67-domain (IQD) proteins have been implicated in both plant defense and organ development [19]. GhIQD10 interacts with GhCaM7 to control cotton fiber elongation via calcium signaling, and the interaction was inhibited by Ca^2+^ [20]. In soybean, GmIQD63 interacts with GmCDPK38 to mediate defense against the common cutworm [21]. Moreover, the consistent upregulation of *PvIQD4*, *PvIQD10*, *PvIQD14*, and *PvIQD32* suggests that IQD genes may also play an important role in the response to salt stress [22]. However, the potential role of TaIQD in wheat salt stress signaling remains largely unexplored.

Wheat (*Triticum aestivum* L.) is one of the most important staple crops worldwide, providing essential calories and nutrients for the global population. However, wheat production is severely threatened by soil salinity, which has become one of the major abiotic stresses limiting crop productivity [23]. Excessive salinity impairs seedling establishment, vegetative growth, reproductive development, and grain yield, ultimately resulting in substantial agricultural losses [24]. According to FAO 2024, more than 1.38 billion hectares of land worldwide are currently affected by salinity [25]. By 2050, global warming and freshwater scarcity will result in over 50% of arable land being affected by salt, saline conditions drastically reduce wheat yields, with losses exceeding 60% compared to non-saline soils [26]. Salt tolerance is a genetically and physiologically complex trait, largely regulated by multiple genes and intricate signaling networks. Unfortunately, most modern wheat cultivars exhibit only limited tolerance to salinity, making them highly vulnerable to yield reductions in salt-affected areas [27]. Therefore, the identification and functional characterization of novel salt-responsive genes is a crucial step toward developing salt-tolerant wheat varieties, which is of the great significance for global food security.

Genome-wide transcriptome profiling of CaM/CMLs provides an effective strategy to identify novel regulators of wheat abiotic stress responses. However, the specific roles of CaM/CMLs in wheat stress adaptation remain poorly understood. This study performed the comprehensive analysis of subgroup II CaM/CMLs, including the phylogenetic relationships, conserved motifs, sequence features, and *cis*-elements analysis. Transcriptome analysis and quantitative real-time PCR (qRT-PCR) analysis revealed that *TaCML49-B* was strongly induced by salt stress in wheat roots. We performed the functional identification and mechanism analysis of *TaCML49-B*. TaCML49-B was localized to nucleus and plasma membrane. Overexpression of *TaCML49-B* in *Arabidopsis* increased the root growth under NaCl treatment; transgenic plants showed less wilting rate and cell membrane damage than WT under salt treatment in soil. To further study the function of *TaCML49-B*, BSMV-VIGS-mediated silencing was performed in wheat, and the silenced plants exhibited reduced salt tolerance and increased membrane damage. The prediction analysis and bimolecular fluorescence complementation assay (BiFC) indicated that TaCML49-B interacted with TaIQD23. Collectively, our results demonstrate that *TaCML49-B* functions as a positive regulator in wheat salt stress response. Our findings advance the understanding of wheat CaM/CMLs function, thereby providing foundational genetic resources for developing salt-tolerant wheat varieties.

## 2. Results

### 2.1. Subgroup II CaM/CMLs Are Involved in Responses to Multiple Abiotic Stresses

**Figure 1:**
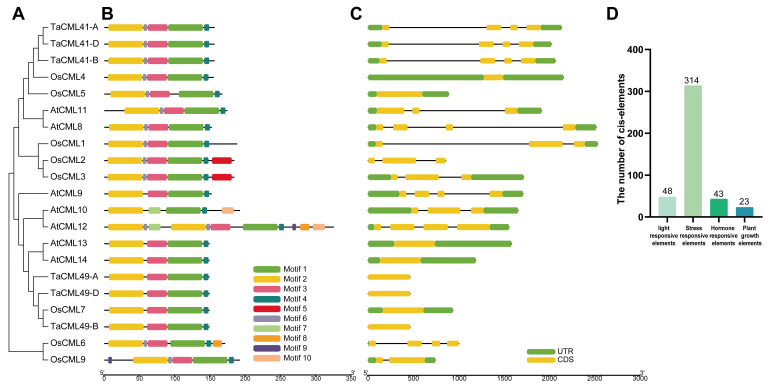
Phylogenetic relationships, conserved motifs, gene structures, and *cis*-elements of subgroup II CaM/CMLs in wheat, rice, and *Arabidopsis*. (**A**) A maximum-likelihood (ML) phylogenetic tree of CaM/CML proteins from wheat (Ta), rice (Os), and *Arabidopsis* (*At*) constructed using MEGA X with 1000 bootstrap replicates. (**B**) Conserved motif distributions of 21 CaM/CMLs. (**C**) Gene structures of 21 CaM/CMLs. (**D**) Predicted *cis*-elements in promoter regions analyzed using the PlantCARE database.

In a previous study, 128 CaM/CMLs were identified in wheat and designated according to their chromosomal locations. Phylogenetic analysis classified these genes into nine subgroups (group I-IX) [18]. Within subgroup II, 21 proteins from wheat, rice, and *Arabidopsis* were identified (Figure 1A, Table S1). Multiple subgroup II genes have been reported to participate in abiotic stress responses in *Arabidopsis* [7,28] and rice [29,30]. In wheat, subgroup II contains six homologous members corresponding to two genes, *TaCML41* and *TaCML49* (Figure 1A).

Gene structure analysis is essential for understanding gene expression and function. To characterize subgroup II members, the 10 most statistically significant conserved motifs were identified, designated as motifs 1–10 (Figure 1B, Table S2). The number of motifs per protein ranged from 4 to 10, and most subgroup II CaM/CMLs exhibited relatively conserved motif compositions. Structural analysis further revealed that the majority of subgroup II CaM/CMLs contained two untranslated regions (UTRs), a single exon, and no introns (Figure 1C, Table S3).

Since gene expression is often regulated by *cis*-elements in promoter regions, we examined the upstream sequences of subgroup II CaM/CMLs and identified a total of 428 potential *cis*-elements (Figure 1D, Table S4). These elements were grouped into four major categories: light-responsive elements (11.21%), hormone-responsive elements (10.04%), environmental stress-responsive elements (73.36%), and plant growth-related elements (5.37%) (Figure 1D). The predominance of stress-related *cis*-elements suggests that subgroup II CaM/CMLs may play key roles in mediating plant responses to abiotic stresses.

**Figure 2:**
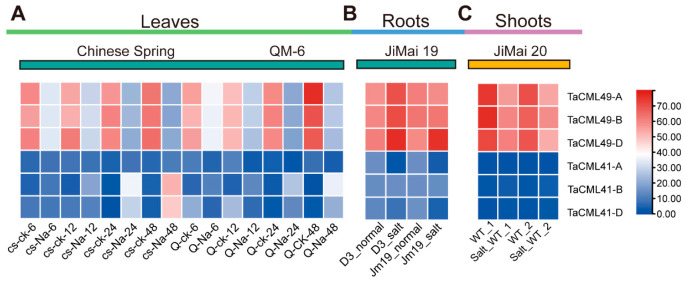
Expression profiles of *TaCML41* and *TaCML49* in different wheat tissues. Transcriptome data from the Expression Atlas were used to analyze transcript accumulation in (**A**) leaves, (**B**) roots, and (**C**) shoots. Heatmaps were generated using EvolView, with transcript levels indicated by color gradients. CS, Chinese Spring; CK, control; QM, Qingmai; Na, salt stress; D3, overexpression of DREB in wheat (Jimai19); Jm, Jimai; WT, wild type.

To investigate the expression profiles of *TaCML41* and *TaCML49*, wheat salt-related transcriptome data (PRJNA293629, PRJNA355905 and PRJNA374931) were analyzed. The expression of *TaCML41* showed no significant variation across tissues or treatment conditions. In contrast, *TaCML49* was downregulated in leaves and shoots (Figure 2A,B), but markedly upregulated in roots (Figure 2C). Based on this root-specific induction, subsequent analyses were focused on *TaCML49-B*.

### 2.2. Expression Pattern Analysis of TaCML49-B Under Salt Stress

**Figure 3:**
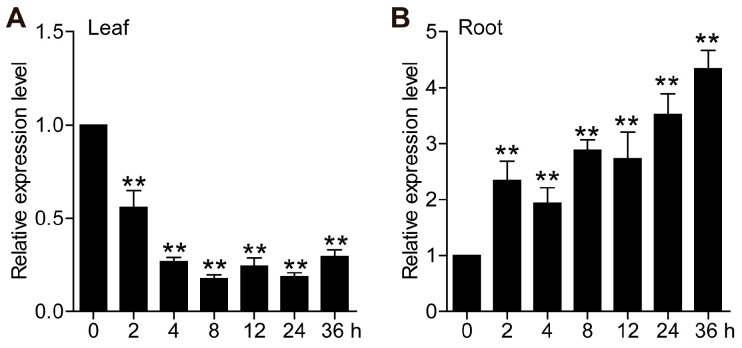
Expression patterns of *TaCML49-B* under salt stress. qRT-PCR analysis of *TaCML49-B* expression in (**A**) leaves and (**B**) roots of seven-day-old wheat seedlings subjected to 200 mM NaCl treatment. Wheat TaActin was used as an internal control. Data represent mean ± SD from three biological replicates. Significant differences were determined by Student’s t-test (** *p* < 0.01).

The expression patterns of *TaCML49-B* under salt stress were examined using qRT-PCR. Samples from leaves and roots were collected at 0 h, 2 h, 4 h, 8 h, 12 h, 24 h, and 36 h after salt treatment. In leaves, the transcript levels of *TaCML49-B* decreased significantly under salt stress (Figure 3A), whereas the levels were markedly upregulated in roots (Figure 3B). These quantitative results were consistent with the transcriptome data, confirming that *TaCML49-B* is involved in salt stress response.

### 2.3. TaCML49-B Was Located to Nucleus, Cytoplasm and Membrane

**Figure 4:**
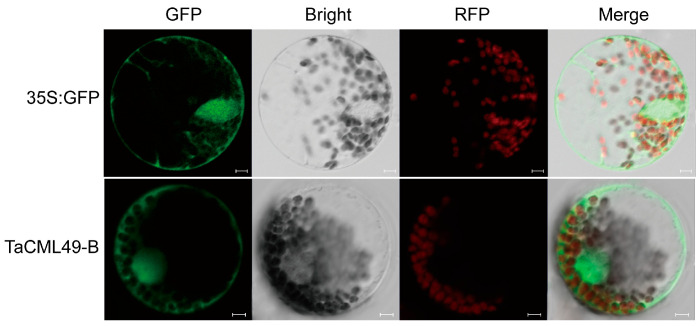
Subcellular localization of TaCML49-B. The p16318hGFP (control) and *TaCML49-B*-GFP recombinant were transiently expressed in wheat protoplasts The green indicates GFP signals, and the red indicates chloroplast autofluorescence. Results were observed after transformation for 18 h with confocal microscopy. Scale bars = 5 mm.

The subcellular localization of *TaCML49-B* was examined in wheat protoplasts leaves using a green fluorescent protein (GFP) fusion construct. Confocal microscopy revealed that TaCML49-B predominant localized to the nucleus, cytoplasm and membrane (Figure 4).

### 2.4. Overexpression of TaCML49-B Enhances Salt Tolerance in Arabidopsis

**Figure 5:**
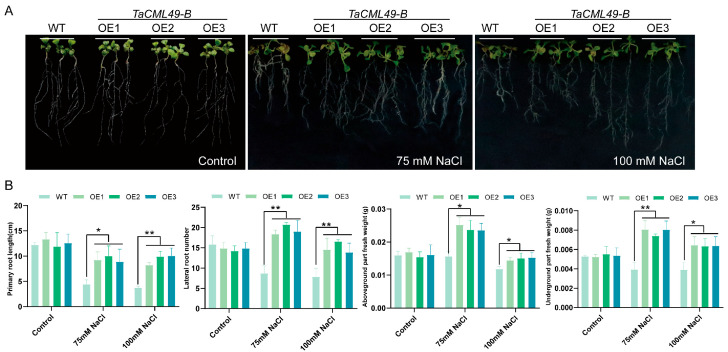
Phenotypic analysis of *TaCML49-B* transgenic *Arabidopsis* under salt stress. (**A**) Root length assays of WT and *TaCML49-B* OE lines on MS medium and MS medium supplemented with 75 mM or 100 mM NaCl. (**B**) The primary root length, lateral root number, shoot fresh weight, and root fresh weight of WT and OE lines. Five-day-old seedlings were transferred to the indicated media and grown for 7 days. Data represent mean ± SD from three biological replicates. Significant differences were determined by Student’s t-test (* *p* < 0.05, ** *p* < 0.01).

To assess the role of *TaCML49-B* in salt-stress tolerance, transgenic lines overexpressing *TaCML49-B* were generated in *Arabidopsis*, and three overexpression (OE) lines with higher levels (OE1, OE2, and OE3) were selected by qRT-PCR. Five-day-old seedlings of wild type (WT) and OE lines were grown on 1/2 murashige and skoog (MS) medium supplemented with 0 (control), 75, or 100 mM NaCl (Figure 5A). After 7 days, primary root length, lateral root number, shoot fresh weight, and root fresh weight were measured (Figure 5B). Under normal conditions, no significant differences were observed between WT and OE lines. In contrast, under NaCl treatment, overexpressing *TaCML49-B* seedlings exhibited longer primary roots, increased lateral root numbers, and higher shoot and root fresh weights compared with WT plants. These results indicate that overexpression of *TaCML49-B* enhances salt-stress tolerance in *Arabidopsis*.

**Figure 6:**
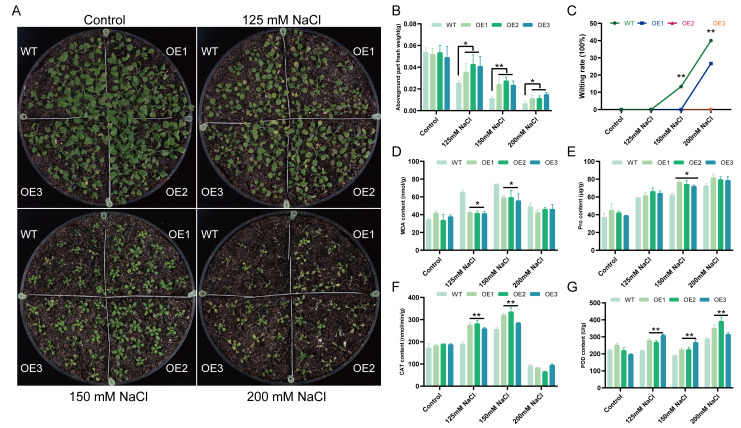
Overexpression of *TaCML49-B* enhances salt tolerance in soil-grown *Arabidopsis*. (**A**) Phenotypes of 21-day-old WT and *TaCML49-B* overexpression plants under 0, 125, 150, or 200 mM NaCl treatments. (**B**) Aboveground fresh weight of WT and OE lines under different salt treatments. (**C**) Wilting rate of WT and OE lines under different salt treatments. (**D**–**G**) Physiological parameters of WT and overexpression plants under normal and salt stress conditions, including MDA (**D**), Pro (**E**), CAT (**F**), and POD (**G**) contents. Data represent mean ± SD from three biological replicates. Significant differences were determined by Student’s t-test (* *p* < 0.05, ** *p* < 0.01).

To assess salt tolerance in soil, 21-day-old WT and *TaCML49-B* OE lines were irrigated with 0, 125, 150, or 200 mM NaCl solutions (Figure 6A). The wilting rate and shoot fresh weight were measured under different salt treatments (Figure 6B,C), and the physiological parameters including malondialdehyde (MDA), catalase (CAT), peroxidase (POD), and proline (Pro) content were analyzed (Figure 6D–G). Under normal growth conditions, no significant differences were observed between WT and OE lines in wilting rate, shoot fresh weight, or physiological parameters. Under different salt treatments, the aboveground fresh weight of OE lines was significantly higher than WT, while the wilting rate of OE lines was lower than WT. Under 125 mM and 150 mM NaCl, OE lines exhibited higher CAT, POD, and Pro contents, whereas MDA levels were lower compared with WT, which revealed that *TaCML49-B* OE lines had less cell membrane damage. These results indicate that overexpression of *TaCML49-B* enhances salt tolerance in *Arabidopsis*.

### 2.5. Silencing of TaCML49-B Alters Salt Response in Wheat

**Figure 7:**
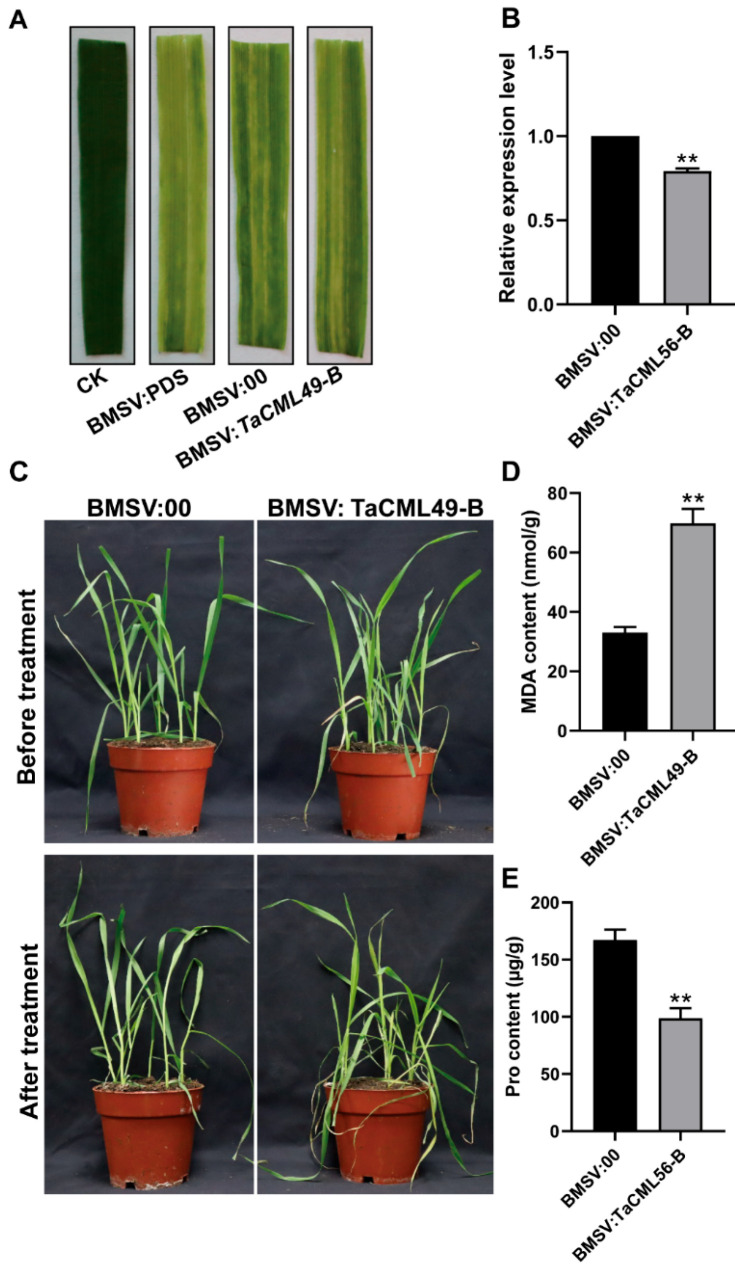
Functional analysis of wheat *TaCML49-B* using BSMV-VIGS. (**A**) Phenotypes of wheat plants following BSMV-VIGS infection. (**B**) qRT-PCR analysis of *TaCML49-B* expression in plants infected with BSMV: *TaCML49-B* or BSMV:00 (control). (**C**) Phenotypic response of BSMV: *TaCML49-B* and control plants under salt stress. (**D**,**E**) Physiological parameters including MDA and Pro contents in BSMV: *TaCML49-B* and control plants under salt stress. Data represent mean ± SD from three biological replicates. Significant differences were determined by Student’s t-test (** *p* < 0.01).

To further investigate the function of *TaCML49-B* in wheat, the BSMV-VIGS (barley stripe mosaic virus–virus-induced gene silencing) technique was employed. A conserved 250-bp cDNA fragment from all three *TaCML49-B* homologs was selected from the coding sequence (CDS) to generate the recombinant virus BSMV: *TaCML49-B*. Ten days after virus inoculation, virus-infected plants exhibited speckled stripes (Figure 7A). qRT-PCR analysis confirmed that *TaCML49-B* expression was significantly reduced in the leaves of BSMV: *TaCML49-B*-infected plants compared with control BSMV:00 plants (Figure 7B). Subsequently, virus-infected wheat plants were subjected to NaCl stress. After seven days, BSMV: *TaCML49-B* plants displayed higher sensitivity to salt stress than BSMV:00 plants (Figure 7C). Phenotype analysis revealed that MDA content was elevated, whereas Pro content was decreased in BSMV: *TaCML49-B* plants compared with controls (Figure 7D,E). These results indicate that silencing of *TaCML49-B* reduces salt resistance in wheat, demonstrating that *TaCML49-B* acts as a positive regulator in salt stress response.

### 2.6. Protein Interaction Network of TaCML49-B

**Figure 8:**
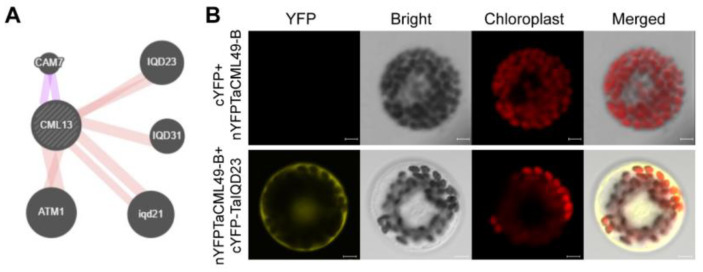
TaCML49-B interact with TaIQD23. (**A**) Predicted protein–protein interaction network of TaCML49-B generated using the STRING database. (**B**) BiFC assay in wheat protoplasts. TaCML49-B coding sequence was fused to the N-terminal half of YFP (pSPYNE, nYFP), and TaIQD23 coding sequence was fused to the C-terminal half of YFP (pSPYCE, cYFP). cYFP and nYFP-TaCML49-B were served as control. Fluorescence signals indicate the interaction between TaCML49-B and TaIQD23. Scale bars = 5 mm.

To predict potential protein interactions, the STRING web server was used. The analysis suggested a possible interaction between TaCML49-B and TaIQD23 (Figure 8A). To further validate this interaction, BiFC assay was performed. *TaCML49-B* fused to the N-terminal half of YFP (nYFP) and *TaIQD23* fused to the C-terminal half of YFP (cYFP) were transiently co-expressed in wheat protoplasts. Yellow fluorescence signals were detected, confirming TaCML49-B interacted with TaIQD23 (Figure 8B).

## 3. Discussion

Plant Ca^2+^ sensor proteins play essential roles in Ca^2+^ signaling networks, maintaining Ca^2+^ homeostasis during diverse cellular processes [31,32]. CaM/CMLs constitute a major group of EF-hand-containing Ca^2+^ sensors in plants, which bind Ca^2+^ and regulate downstream targets in response to stimulus-induced Ca^2+^ fluctuations and signal transduction [33]. Although 128 TaCaM/CMLs have been classified into nine subgroups in wheat [18], functional data on these genes remain limited. In the present study, a comprehensive analysis of Subgroup II CaM/CMLs from wheat, rice, and *Arabidopsis* was performed, including phylogenetic relationships, conserved motifs, and gene structures. Furthermore, *TaCML49-B* was significantly upregulated in roots under salt stresses and was, thus, selected for functional identification and mechanism analysis.

A phylogenetic analysis was performed to compare CaM/CMLs from wheat, *Arabidopsis*, and rice (Figure 1). The results indicated that both sequence and function of CaM/CMLs are largely conserved among these species. However, wheat CaM/CMLs exhibited a closer phylogenetic relationship with rice than with *Arabidopsis* (Figure 1). Moreover, although differences in intron-exon structures were observed among the 21 CaM/CMLs, members within the same phylogenetic branch shared common structural characteristics (Figure 1). These findings suggest that the combination of conservation and diversity in motifs and gene structures contributes to the evolution and functional diversification of this gene family.

Abiotic stresses activate genes critical for stress resistance. Numerous stress-responsive *cis*-elements (CREs) were identified in the promoter regions of the 21 CaM/CMLs analyzed, indicating potential roles in plant stress responses. For instance, *AtCML9* expression in seedlings is rapidly induced by abiotic stress and abscisic acid (ABA) [34]. RNA-seq analysis highlighted regulatory relationships between *CML8* and genes involved in growth and brassinosteroid (BR) signaling, and co-immunoprecipitation experiments demonstrated that *CML8* interacts with the BR receptor BRI1 in a ligand-dependent manner [35]. Additionally, RNA interference of *AtCML13* and *AtCML14* in mature plants resulted in shortened siliques, reduced root systems, and accelerated leaf senescence [28].

Subgroup II of CaM/CMLs includes six homologous genes corresponding to two wheat genes (*TaCML41* and *TaCML49*), the functions of which in wheat have not been fully characterized. In the present study, transcript levels of these six homologs were analyzed in three wheat tissues at different developmental stages using previously reported transcriptome data. Transcript accumulation of *TaCML41* and *TaCML49* (Figure 2), together with qRT-PCR results, indicated that *TaCML41-B* is responsive to salt stress and may regulate plant responses to salinity. Interestingly, *TaCML49-B* was downregulated in leaves but upregulated in roots under salt stress, indicating tissue-specific regulation (Figure 3). Such contrasting expression patterns suggest that *TaCML49-B* may function not only in salt-stress signaling but also in processes related to stress-induced aging or senescence. Consistently, in *Oryza sativa*, *OsCML4* is highly expressed under 150 mM NaCl stress [30]; as *TaCML41* is homologous to *OsCML4*, it is likely involved in salt and drought stress responses in wheat.

To further investigate *TaCML41-B* function under salt stress, transgenic *Arabidopsis* overexpressing *TaCML41-B* was generated. *TaCML41-B* remained relatively insensitive to 100 mM NaCl, whereas WT plants showed a hypersensitive phenotype (Figure 5), suggesting that *TaCML41-B* is crucial for salt tolerance. Physiological analyses showed that, under salinity, MDA content was significantly reduced, whereas Pro content was significantly elevated in *TaCML41-B* overexpressing plants (Figure 6). These results indicate that overexpression of *TaCML41-B* enhances salt tolerance in *Arabidopsis*. Conversely, silencing of *TaCML41-B* in wheat using BSMV- VIGS led to decreased salt tolerance, accompanied by increased MDA content and decreased Pro levels (Figure 7). As MDA reflects lipid peroxidation and membrane injury and Pro functions as both an osmoprotectant and ROS scavenger, these changes suggest that *TaCML41-B* enhances salt tolerance by alleviating oxidative damage and maintaining cellular homeostasis.

**Figure 9:**
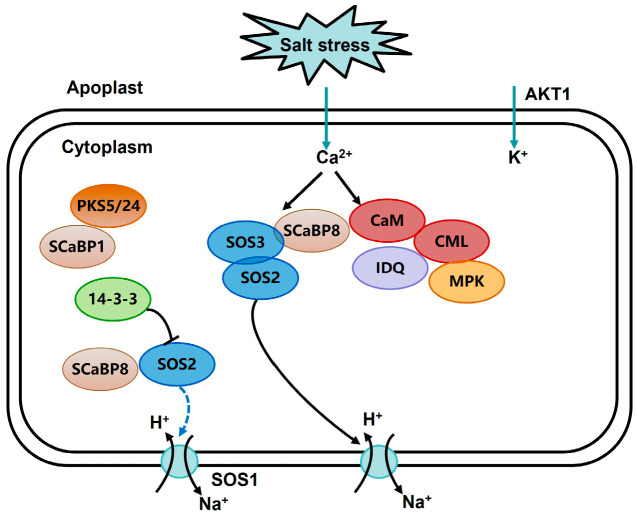
Schematic diagram of salt-stress response, including CML regulation.

By predicting the interaction network of TaCML41-B, it was found that TaCML41-B interacts with TaIQD23 (Figure 8). Previous studies have reported that *Arabidopsis* IQD1 can interact with CaM/CMLs in vitro in a Ca^2+^-dependent manner [36]. Moreover, two IQD genes in cotton, *Gh_S06G0014* and *Gh_S09G1608*, were shown to enhance tolerance to drought and salt stress [37]. These findings suggest that the interaction between TaCML41-B and TaIQD23 may contribute to improved salt-stress tolerance in wheat (Figure 9). Collectively, our results indicate that *TaCML41-B* functions as a positive regulator of salt-stress tolerance, underscoring its important role in wheat responses to abiotic stress.

## 4. Materials and Methods

### 4.1. Sequence Characteristics and Phylogenetic Analysis of 21 CaM/CMLs

The nucleic acid and protein databases of 21 CaM/CMLs of *Arabidopsis*, rice, and wheat were downloaded from the Ensemble Plants database (http://plants.ensembl.org/index.html, accessed on 2 June 2025). The CaM/CMLs protein sequences were aligned using ClustalX 2.0 with the default parameters [38]. A phylogenetic tree was constructed using the maximum likelihood method with 1000 bootstrap replicates as implemented in MEGA X [39].

### 4.2. Exon-Intron Structure, Motif Analysis and Cis-Elements Analysis of 21 CaM/CMLs

The 21 CaM/CMLs gene information was extracted from the GFF3 file and the information was visualized using the gene structure plate in TBtools software V2.326 [40]. The wheat gene protein sequences were integrated, the conserved motifs were analyzed by the online tool MEME (http://alternate.meme-suite.org/tools/meme, accessed on 1 August 2025) [41], and the predicted results were put into TBtools software for visualization.

To analysis the possible biological functions and transcriptional regulation of 21 CaM/CMLs. The 2.0 kb region sequences upstream from start codons were downloaded from Ensemble Plants database and then submitted to PlantCARE database for *cis*-elements analysis (http://bioinformatics.psb.ugent.be/webtools/plantcare/html/ (accessed on 2 August 2025)).

### 4.3. Expression Patterns Analysis of TaCML41 and TaCML49

The expression data of *TaCML41* and *TaCML49* gene in leaves and roots after salt stress were downloaded from the abiotic stress transcriptome. The abiotic stress transcriptome data can be searched from the NCBI website (https://www.ncbi.nml.nih.gov, accessed on 3 August 2025). TBtools software is then used to visualize the data obtained above [42].

### 4.4. Plant Material and Stress Treatments

We examined the expression patterns of candidate genes in wheat variety ‘Fielder’. The seeds were treated with H_2_O_2_ and transferred for 4 °C to break dormancy. After germination, the seeds were transferred to a hydroponic box for 25 °C cultivate with photoperiod of 16/8 h. The root and leaf tissues of wheat plants were collected at 0 h, 2 h, 4 h, 8 h, 12 d, 24 d, and 36 h after 200 mM NaCl treatment. The collected samples were immediately frozen in liquid nitrogen and stored in a −80 °C refrigerator for RNA extraction.

### 4.5. RNA Isolation and Quantitative Real-Time PCR (qRT-PCR)

RNA was extracted from wheat using the Novozan kit, and the first strand cDNA was synthesized using the full-type Gold Company kit during reverse transcription. ABI Prism 7500 real-time PCR system (Applied Bio systems, Foster City, CA, USA) was used for qRT-PCR and the reagents of Vazyme Taq Pro Universal SYBR qPCR Master Mix. Gene cluster-specific and internal reference was conducted using gene *TaActin* (accession no. AB181991). The qRT-PCR program was carried out as follows: pre-denaturation at 95 °C for 30 s; denaturation at 94 °C for 10 s, annealing at 60 °C for 30 s, extension at 72 °C for 34 s, 40 cycles. At least three biological replicates were used for qRT-PCR analysis, and the 2^−ΔΔCt^ method was used to analyze the data [43]. The amplification efficiency of primers was determined by melting curve analysis. Primers were listed in Table S5.

### 4.6. Subcellular Localization of TaCML49-B

The full-length coding sequence (CDS) of *TaCML49-B* with the stop codon removed was cloned into p16318hGFP vector under control of the CaMV35S promoter. For transient expression assays, 7-day-old wheat seedlings were used for the isolation of wheat protoplasts. The p16318hGFP-TaCML49-B and control plasmids were transformed into wheat protoplasts mediated by PEG4000, as described by [44]. The transfected protoplasts were incubated at 22 °C for 18 h in darkness, after which GFP signals were observed with 488 nm and 543 nm illumination by a confocal laser scanning microscopy (LSM700, CarlZeiss, Oberkochen, Germany). Primers were listed in Table S5.

### 4.7. Transformation of Arabidopsis and Propagation of Positive Seedlings

The CDS of *TaCML49-B* was cloned into the vector pCAMBIA2300 and the recombinant vector 2300-*TaCML49-B* was obtained. The correct recombinant vector was transformed into *Agrobacterium tumefaciens* strain GV3101. Transgenic *Arabidopsis thaliana* was obtained by *Agrobacterium*-mediated inflorescence method [45]. Columbia-0 (WT) was used as control. T_0_ generation transgenic seeds were placed in 1/2 MS medium supplemented with 50 mg/L kanamycin to screen positive seedlings until to T_3_ generation.

### 4.8. Salt-Stress Assays of Transgenic Arabidopsis Plants

Three homozygous T_3_ lines were selected for phenotypic analysis. For the root assay, WT and transgenic seeds were disinfected and vernalized for 3 days to break dormancy. After 5 days of growth, WT and transgenic *Arabidopsis* plants were transplanted to 1/2 MS solid medium with different concentrations of NaCl (75, and 100 mM) for salt-stress treatment. After 7 days of treatment, *Arabidopsis* seedlings were scanned using the Epson Expression 11000XL flatbed scanner (Epson, Suwa, Japan) to determine the length of taproot and number of lateral roots. Then samples were taken to determine the fresh weight of aboveground and underground *Arabidopsis* seedlings after treatment.

In the phenotypic observation experiment, seeds of WT and transgenic *Arabidopsis* plants were seeded into flowerpots and salt-stress tests were carried out when they were 3 weeks old. The flowerpots were watered with different concentrations of NaCl solution (125 mM, 150 mM, and 200 mM NaCl). After 7 days of salt treatment, the phenotypes were observed, and the wilting rate and fresh weight of above-ground parts were calculated. The contents of MDA, PRO, CAT, and POD in transgenic *Arabidopsis* seedlings treated with salt solution at different concentrations were detected with the physiological index assay kit (Suzhou Comin Biotechnology, Suzhou, China). The test was carried out according to the determination method in the kit instructions, and three biological replicates were performed for each experiment.

### 4.9. BSMV-VIGS-Induced TaCML49-B Silencing Assays

A conserved 200–300 bp cDNA fragment was isolated from *TaCML49-B* using the online tool SGN VIGS (https://vigs.solgenomics.net/, accessed on 3 July 2025). The γ-*TaCML49-B* was sequenced by inserting it into a γ plasmid with the restriction site *Nhe* I. The α, β, and γ plasmids (γ-*TaCML49-B* and γ-GFP) were linearized, respectively. The restriction sites of α and γ plasmids were *Mul* I, and the restriction sites of β plasmids were *Spe* I. RNA was synthesized according to the instructions of the Ribo MAX™Large Scale RNA Production System-T7 kit (Promega, Fitchburg, WI, USA). The synthesized RNA-α, RNA-β, RNA-γ- *TaCML49-B* (or control RNA-γ-GFP) were mixed and friction-inoculated into two-leaf wheat leaves. Ten days after virus transfection, the leaves were collected to monitor the virus infection [46,47]. The expression level of *TaCML49-B* in the leaves after inoculation was detected using qRT-PCR. Then, the silenced plants were treated with salt stress to observe the changes of phenotype and relative expression.

### 4.10. Prediction and Validation of TaCML49-B Interaction Networks

Protein interaction networks of the TaCML49-B were predicted using the STRING database (https://cn.string-db.org/ (accessed on 10 August 2025)).

### 4.11. Bimolecular Fluorescence Complementation (BiFC) Assay

The vectors used in the BiFC experiment are pSPYNE (nYFP) and pSPYCE (cYFP). The BamHI restriction enzyme from the NEB company was used to cleave nYFP and cYFP vectors. The design adapter was primed, the stop codons were removed, and the target gene with an adapter from the constructed zero background vector was amplified. Construct nYFP-*TaCML49-B* and nYFP-*TaIQD23* vectors. Extract plasmids from the correctly sequenced bacterial solution, extract wheat protoplasts, and add two types of plasmids (10 μg each) to verify their interaction, co-transformed cYFP and nYFP-*TaCML49*-B constructs as control. Observe the yellow fluorescence signal under a confocal laser microscope. The sequences for the primers used are listed in Table S5.

## 5. Conclusions

In this study, a comprehensive analysis of Subgroup II of the CaM/CMLs was conducted, including characterization of sequence features, phylogenetic relationships, conserved motifs, and *cis*-elements. To investigate the molecular mechanisms underlying abiotic stress tolerance, the function of a key CML protein, TaCML49-B, was characterized. Gene expression analyses indicated that *TaCML49-B* may play important roles in both development and stress responses. Overexpression of *TaCML49-B* in *Arabidopsis* enhanced tolerance to salt stress, whereas silencing of *TaCML49-B* in wheat reduced salt tolerance, demonstrating that *TaCML49-B* functions as a positive regulator of salt-stress responses. Furthermore, the interaction between TaCML49-B and TaIQD23 was confirmed, supporting its involvement in wheat stress-response pathways. Collectively, these findings provide comprehensive insights into the TaCML gene family in wheat and offer a foundation for further functional characterization aimed at developing stress-resistant wheat varieties.

## Data Availability

Data Availability Statement: Data are contained within this article and Supplementary Materials.

## Supplementary Materials

The following supporting information can be downloaded at: https://www.mdpi.com/article/10.3390/plants14203163/s1, Table S1: List of sequence IDs used in the phylogenetic analysis; Table S2: List of conserved motifs of 21 CaM/CMLs proteins; Table S3: Exon and intron numbers of 21 CaM/CMLs; Table S4: *Cis*-elements in the promoter region of 21 CaM/CMLs; Table S5: The primers used in this study.
